# Case Report: Angiomatoid fibrous histiocytoma in the hand: a rare clinical presentation and diagnostic challenge

**DOI:** 10.3389/fonc.2023.1280630

**Published:** 2023-12-12

**Authors:** Jiro Ichikawa, Tomonori Kawasaki, Hiroki Imada, Masanori Wako, Taro Fujimaki, Rikito Tatsuno, Takahiro Jubashi, Hirotaka Haro

**Affiliations:** ^1^ Department of Orthopaedic Surgery, Interdisciplinary Graduate School of Medicine, University of Yamanashi, Chuo, Japan; ^2^ Department of Pathology, Saitama Medical University International Medical Center, Hidaka, Japan; ^3^ Department of Pathology, Saitama Medical Center, Saitama Medical University, Kawagoe, Japan

**Keywords:** angiomatoid fibrous histiocytoma, magnetic resonance imaging, histopathology, differential diagnosis, clinical presentation

## Abstract

Angiomatoid fibrous histiocytoma (AFH) is a rare tumor of mesenchymal origin occurring in young adults. Based on its clinical course, it is said to have an intermediate potential. We present a case of a 59-year-old woman with AFH in the hand that was difficult to diagnose. A benign soft tissue tumor was suspected on magnetic resonance imaging, and its size and open biopsy suggested nodular fasciitis or inflammatory myofibroblastic tumor. A diagnosis of AFH was eventually made based on the analysis of the resected specimens. The characteristic findings of histopathology and immunohistochemistry in this case were relatively poor, so fluorescence *in situ* hybridization contributed to making the correct diagnosis. Considering its prognosis, careful follow-up was decided upon without additional surgery. Our case is a challenging one because of its atypical presentation and inconclusive imaging and histopathological findings.

## 1 Introduction

Angiomatoid fibrous histiocytoma (AFH) is a rare soft tissue neoplasm, accounting for approximately 0.3% of all soft tissue tumors, with an intermediate potential (1). AFH frequently occurs in the extremities (1); however, there were only 7 cases reported in the hand and fingers (2–4). Although a wide age range has been reported, AFH occurs in the first two decades (1). The characteristic imaging findings of AFH have been reported (5); however, histopathological findings are essential for correct diagnosis. Unfortunately, making a diagnosis is difficult in some cases, even with histopathology, because there is no specific immunohistochemistry (IHC) marker; therefore, fluorescence *in situ* hybridization (FISH) has been a helpful tool in making the diagnosis (6). Herein, we present a challenging case of AFH in a rare location and with diagnostic difficulty.

## 2 Case description

**Figure 1:**
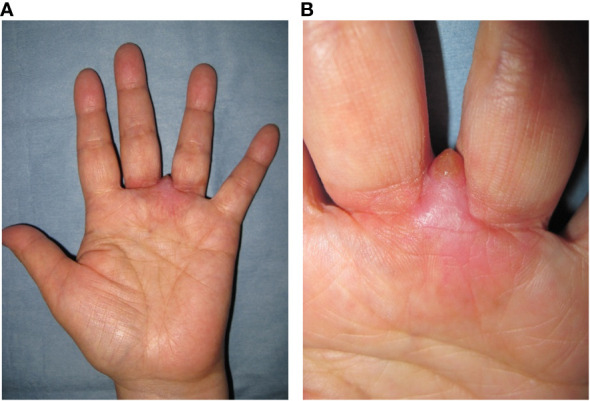
Photograph at first visit. Soft tissue mass with redness located between the middle and ring finger **(A, B)**.

**Figure 2:**
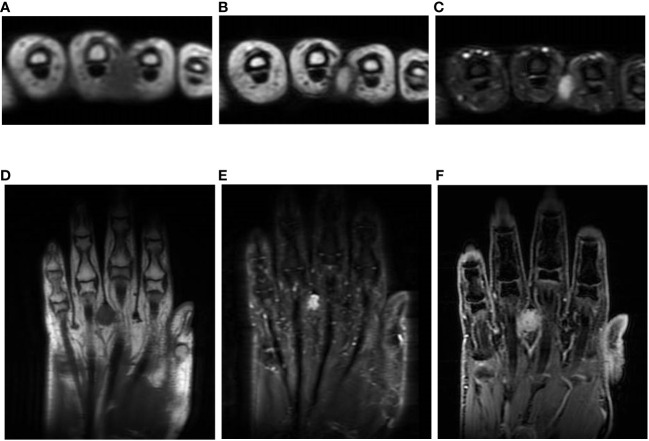
Imaging findings. T1-weighted axial images showing low signals **(A, D)**; homogeneous high signal on T2-weighted images **(B)** and short tau inversion recovery images **(C, E)**; and axial and central enhancement on a gadolinium-enhanced T1-weighted image **(F)**.

A 59-year-old woman presented with a mass situated between her right middle finger and ring finger. The mass had been apparent for 1 year and was slightly painful and erythematous, and gradually increased in size (**Figures 1A, B**). Physical examination revealed no bleeding or numbness. Moreover, the tinel sign was negative and the range of motion of the middle and ring fingers was normal. Magnetic resonance imaging (MRI) revealed a low-intensity signal on T1-weighted images (**Figures 2A, D**) and high signal intensity on both T2-weighted images (**Figure 2B**) and T2 short tau inversion recovery images (**Figures 2C, E**). Homogeneous enhancement was noted on gadolinium-enhanced T1-weighted images (**Figure 2F**).

## 3 Diagnostic assessment, treatment, and outcome

**Figure 3:**
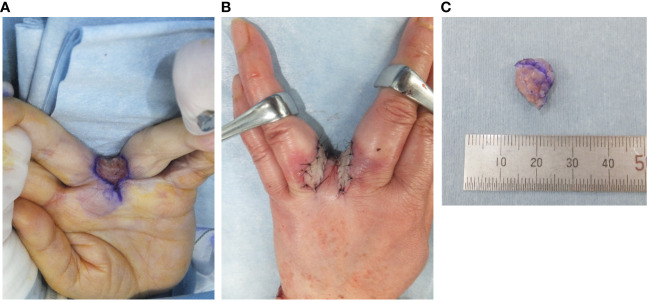
Intraoperative findings and gross specimen. The tumor was resected on the surface of the skin and the defect was covered with a local skin flap and skin graft **(A, B)**. Macroscopically, the tumor measured 1.2 × 1.0 × 0.9 cm in size **(C)**.

**Figure 4:**
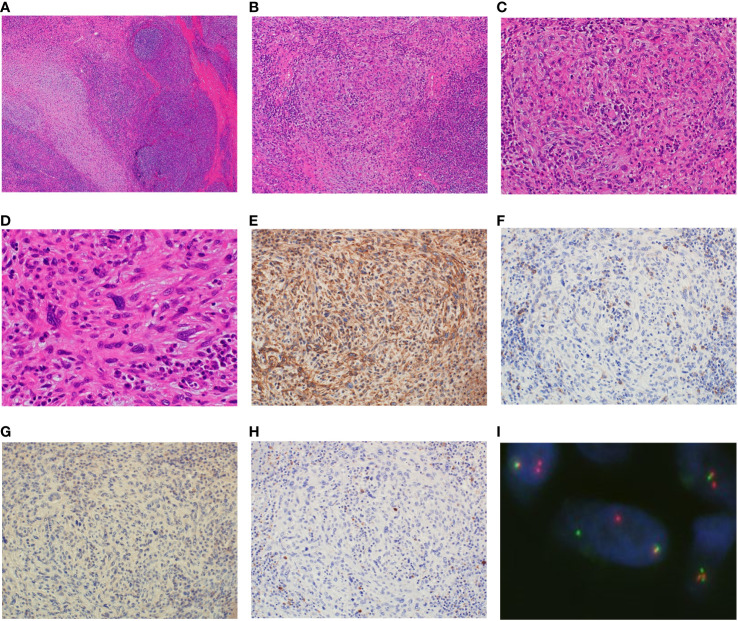
Analysis of the resected specimen. The resected specimen showing the tumor surrounded by foci of lymphoplasmacytic cuffs, and a pseudocapsule and multiple heterogeneous spindle cells showing nuclear atypia without mitotic activity along with large pleomorphic cells (**A**, ×40 **B**, ×100 **C**, ×200 **D**, ×400). Immunohistochemical results for CD99 **(E)**, EMA **(F)**, ALK **(G)** and CD68 **(H)** (×200). Fluorescence *in situ* hybridization showing the EWSR1 split signal **(I)**.

Because making the correct diagnosis based on only the clinical and imaging findings was difficult, we decided to perform an open biopsy. The initial diagnosis was nodular fasciitis or inflammatory myofibroblastic tumor (IMT). Therefore, we recommended surgery several times but the patient declined it because of her busy work schedule. After careful observation and because of the minor symptoms and small size for 6 years, she accepted to undergo surgery after she had eventually retired. After considering the biopsy diagnosis and the maintenance of finger function, a marginal resection with skin on the surface of the tumor was performed and the interdigit defect was covered with a skin flap and skin graft (**Figures 3A, B**). Macroscopically, the tumor measured 1.2×1.0×0.9 cm in size and was grayish-white in appearance (**Figure 3C**). Histopathologically, the tumor was surrounded by foci of lymphoplasmacytic cuffs and a hyalinized collagen fibrous pseudocapsule, whereas pseudoangiomatous spaces were not identified. Heterogeneous spindle mesenchymal cells showing nuclear atypia in the background of edematous or collagenous fibrous stroma, but lacking mitotic activity, proliferated alongside large pleomorphic cells intermingled with some storiform to irregular multinodular forms (**Figures 4A-D**). IHC indicated that the tumor was weakly positive for CD99 (**Figure 4E**), focally and slightly positive for EMA (**Figure 4F**), and negative for ALK (**Figure 4G**), CD68 (**Figure 4H**), AE1/AE3, CD34, desmin, SMA, and S-100 (data not shown). The Ki67 (MIB-1) labeling index was less than 5% (data not shown). FISH showed a positive *EWSR1* split signal (**Figure 4I**). Taken together, a final diagnosis of AFH was made. We considered additional resection; however, the patient declined additional surgery, and the disease was followed up with both MRI for recurrence and computed tomography for metastasis every 6 months. There was no recurrence at the 1-year follow-up.

## 4 Discussion

AFH is classified as an intermediate tumor because it rarely metastasizes and usually follows a favorable clinical course. It frequently occurs in children and young adults. Regarding the tumor location, the most common site is the extremities, followed by the trunk and the head and neck; there is currently no established pattern between the upper and lower extremities (1, 3, 7). Although AFH is often found in superficial locations (1), the occurrence rate in deep sites was reported as 32–43% (2, 3). Its average size was from 2 to 4 cm (1, 7). To our knowledge, there are only 7 reported cases of AFH in the hand or fingers (2–4). Among these, the average age was 36.4 (8–60) years and the average size was 2.1 cm (1–4.5). Considering these cases, our case is atypical, and we suspected it to be a benign tumor.

Although the double rim sign, marginal neoplastic infiltration, multilocular areas, and fluid-fluid level on MRI were reported as the characteristic findings in AFH (2, 5), the sensitivity and positivity of these findings are still unclear, and moreover, all these features were negative in our case, so further studies are needed. Enhancement was seen in all cases, but the pattern of enhancement varied (2, 5). In addition, the average size was so small, similar to our case, and the differentiation of characteristic findings on MRI seemed to be limited and difficult. In a previous report, the differential diagnosis by MRI was broad, from benign to malignant tumors (2), and moreover, hemangioma and neurogenic tumor were suspected in our case, hence making the correct diagnosis based on MRI seemed impossible.

The histopathological finding of AFH is characterized by a circumscribed and lobulated nodular proliferation of epithelioid to spindle-shaped tumor cells arranged in a syncytial pattern. The tumor cells exhibit moderately eosinophilic and atypical vesicular nuclei (1). Pseudoangiomatous change is a hallmark feature, but it was not observed in approximately 33% of cases similar to ours (6). Moreover, a thick fibrous pseudocapsule with hemosiderin deposition and a pericapsular lymphoplasmacytic rim are frequently observed (1). These distinct histopathological characteristics are diagnostically significant; however, they are not specific and AFH has a wide morphological spectrum, from myxoid to fibrous, making diagnosis more difficult (6). No specific IHC marker for AFH has been identified yet (6). Although markers related to skeletal muscle, including myogenin and MyoD1, as well as S-100 and CD34, were negative in all cases, EMA, CD99, CD68, and desmin were reported as positive; however, the positivity rate for these markers was reported to be only approximately 50% (1, 6, 7).

Accurate diagnosis of AFH is critical due to its variable morphologic features and lack of specific immunophenotypic markers with possible misdiagnosis. The differential diagnoses include inflammatory myofibroblastic tumor (IMT), aneurysmal benign fibrous histiocytoma, follicular dendritic cell sarcoma, and metastatic tumors of lymph nodes (6). IMT, which was initially a misdiagnosis in this case, can be distinguished by the presence of distinct cell borders and vesicular nuclei with nucleoli. Inflammatory cells are also intimately intermingled with neoplastic cells without forming a peritumoral rim. Furthermore, the staining pattern for actin, desmin, and ALK is variable, and the percentage of positivity for desmin in AFH, although negative in our case, was approximately 50% in other reported cases (7). Other important features for the differential diagnosis of aneurysmal benign fibrous histiocytoma, follicular dendritic cell sarcoma, and metastatic tumors of lymph nodes are as follows: i) more heterogeneous cell population than AFH, ii) lack of surrounding lymphocytes, in aneurysmal benign fibrous histiocytoma, i) positivity for CD21 and CD35, in follicular dendritic cell tumor, as well as i) presence of native nodal tissue in metastatic tumors of lymph nodes. In addition, myxoid variant AFH was often very difficult to differentiate from myxoid tumors, including myxoid liposarcoma, extraskeletal myxoid chondrosarcoma, and myxofibrosarcoma (3). Interestingly, five of the seven cases involving the hand were of myxoid variants.

Recently, to aid in diagnosis, FISH has been reported to detect three types of fusion genes, including *EWSR1*-*CREB1*, *EWSR1*-*ATF1*, and *FUS-ATF1*, which have been reported in AFH (6, 8). Moreover *EWSR1*-*CREB1* was seen in more than 90% of cases. In our case, the majority of IHC was negative and FISH was very helpful in making a correct diagnosis; however, *EWSR1*-*CREB1* and *EWSR1*-*ATF1* were not specific to AHF, suggesting that not only FISH but also histopathological findings were carefully examined in diagnosis (6).

Surgery by wide resection was the only treatment option because the effectiveness of chemotherapy and radiotherapy remains unclear (2, 4). Wide resection is recommended but, in some cases, including ours, marginal resection was performed (3, 7). In such cases, the necessity of additional resection is currently unclear and further studies are needed. The recurrence rate was reported as 10–20%, metastasis as less than 5%, and mortality as less than 1% (1, 7, 9). Taken together, even if recurrence and metastasis occurred, it seemed resectable lesions may be treated with surgery. Careful follow-up is essential not only in marginal resection like in our case but also in wide resection.

Here, we present a challenging case of AFH in the hand. Because there is a limitation in diagnosing AFH by MRI, histopathological findings, especially by FISH, have a great advantage in making the correct diagnosis. Since we performed marginal resection, careful follow-up was needed.

## Data availability statement

The original contributions presented in the study are included in the article/supplementary material. Further inquiries can be directed to the corresponding author.

## Ethics statement

Written informed consent was obtained from the individual(s) for the publication of any potentially identifiable images or data included in this article.

## Author contributions

JI: Conceptualization, Data curation, Investigation, Writing – original draft, Writing – review & editing. TK: Conceptualization, Data curation, Investigation, Writing – original draft, Writing – review & editing. HI: Conceptualization, Data curation, Investigation, Writing – original draft, Writing – review & editing. MW: Writing – original draft, Writing – review & editing. TF: Writing – original draft, Writing – review & editing. RT: Writing – original draft, Writing – review & editing. TJ: Writing – original draft, Writing – review & editing. HH: Writing – original draft, Writing – review & editing.
